# Successful Implantation of HeartMate3 in a Small Child After Multimodality Imaging Pathway to Assess Feasibility

**DOI:** 10.1097/MAT.0000000000002069

**Published:** 2023-10-19

**Authors:** Enrico G. Italiano, Francesco Bertelli, Irene Cao, Raffaella Motta, Giovanni Di Salvo, Vladimiro Vida, Massimo A. Padalino

**Affiliations:** From the * Pediatric and Congenital Cardiac Surgery Unit, Department of Cardiac, Thoracic, Vascular Sciences and Public Health, University of Padua, Padua, Italy; † Department of Medicine, Institute of Radiology, University of Padua, Padua, Italy; ‡ Pediatric Cardiology Unit, Department of Women’s and Children’s Health, University of Padua, Padua, Italy.

**Keywords:** left ventricular assist device, pediatric, small thorax, virtual reality, heart transplant

## Abstract

The current use of intracorporeal left ventricular assist devices in children is still limited by small body dimensions. Many children weighing of less than 30 kg requiring durable mechanical circulatory support are implanted with the Berlin Heart EXCOR, a paracorporeal device. We present the case of a girl aged 10 years with a body surface area of 1.01 m^2^ undergoing a safe and effective HeartMate3 implantation despite extremely small thoracic dimensions. Using computed tomography-derived three-dimensional (3D) reconstruction, it was possible to simulate several device positions finding the best HeartMate3 lodging. Simulation-guided pump placement was then obtained in the operating room. Normal HeartMate3 functioning was registered until heart transplant. Our experience shows that preoperative planning and virtual fitting simulation can be effective to assess safety of HeartMate3 implantation even in small children. The 3D reconstruction and simulation may help to increase the pool of children candidates for this device, even though a larger experience is needed to assess the risk profile of the HeartMate3 in such small patients.

Current treatment for end-stage heart failure in children also includes the use of left ventricular assist devices (LVADs). Pediatric patients have estimated mortality as high as 15–30% while on the waiting list for a heart donor.^1^ In this scenario, LVAD as a bridge to transplant can significantly improve survival.^2^ The most frequently used LVAD in children is EXCOR (Berlin Heart GmbH, Berlin, Germany),^3^ a paracorporeal pulsatile device that can be implanted in children of any dimension, despite the rate of complications is not negligible.^3^ The only currently available continuous-flow intracorporeal device is the HeartMate3 (HM3; Abbott, Chicago, IL), with reported improved outcomes in adults. As a general indication, the HM3 implantation is recommended in patients with a body surface area (BSA) of greater than 1.2 m^2^ or weighing of more than 30 kg,^4,5^ because a small thoracic cavity (as in children) has the potential risk of internal chest wall damage caused by LVAD friction on muscles and the rib cage. Also, the risk of thromboembolic event is unknown in this population.

Herein, we report the case of a child with a BSA of 1.01 m^2^, who underwent successful HM3 implantation after three-dimensional (3D)-guided surgical planning to assess implantation feasibility.

## Case Report

A girl aged 10 years (body weight 25 kg, BSA 1.01 m^2^) with a history of heart failure was emergently admitted to the pediatric intensive care unit for epigastric discomfort and hypotension. Increased N-terminal pro b-type natriuretic peptide (NT-proBNP) plasma levels were found (7,842 ng/L, normal values of less than 125 ng/L). INTERMACS class was 3 at admission.

Patient’s medical history was relevant for a diagnosis of MYH7 (gene related cardiomyopathy)-linked dilated cardiomyopathy when she was 6 months old. Ten days before admission, she had severe acute respiratory syndrome coronavirus 2 (SARS-CoV2) infection with mild symptoms. Considering her recent viral infection, it was necessary to rule out SARS-CoV2-related acute myocarditis on chronic heart disease. On admission, transthoracic echocardiography showed left ventricular ejection fraction of 18–20% and severe atrioventricular valves regurgitation. Right ventricular systolic function was normal. Cardiac magnetic resonance showed severe left ventricular (LV) dilatation (indexed end-diastolic volume of 180 ml/m^2^) and did not show any sign of inflammation, ruling myocarditis.

The patient could not be weaned from milrinone and adrenaline infusions. On the contrary, she worsened to INTERMACS class 2. Considering the usual long transplant waiting list in children, she was urgently scheduled for LVAD implantation.

## Management

**Figure 1.**
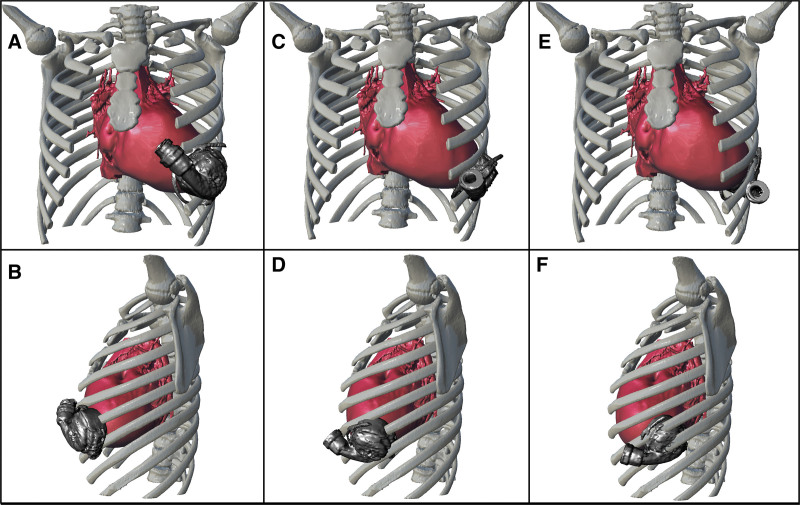
LVAD pump implant simulations with three different positions (unadjusted for LV volume reduction). **A, B:** Anterior placement. **C, D:** Lateral placement. **E, F**: Posterior placement. LV, left ventricle; LVAD, left ventricle assist device.

After 3D chest cavity reconstruction, several 3D virtual HM3 implant simulations were performed to define the spatial encumbrance of the device and its relationship with the rib cage (Figure 1). In dilated cardiomyopathy, the LV cavity shape can be approximated to a sphere. It is known that after LVAD implantation of the LV end-diastolic volume can be reduced up to 50%.^6^ The postoperative LVAD lodging was evaluated applying this information to the preoperative simulations, and the interaction in Figure 1, E and F (*i.e.*, the posterior placement), was chosen for surgery because it was the only one that returned satisfactory results.

### Surgery

**Figure 2.**
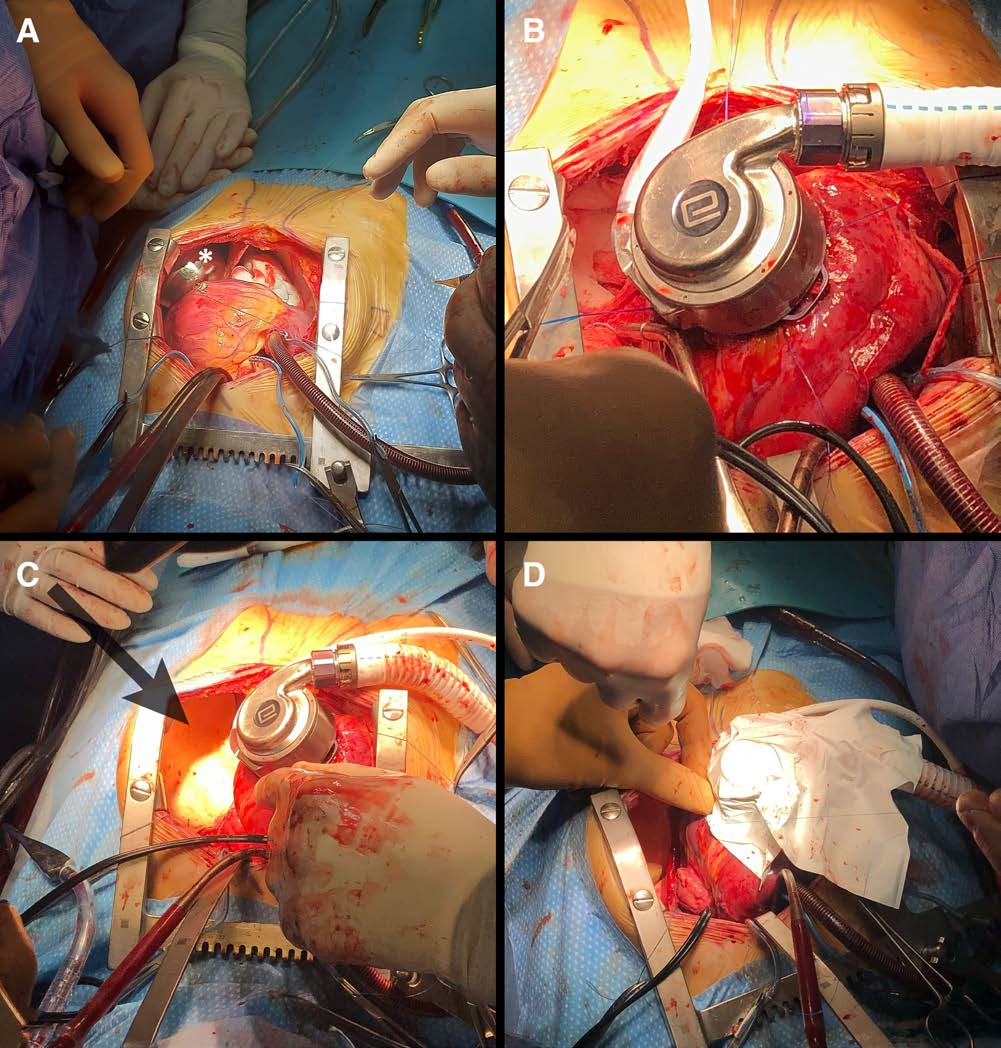
Intraoperative view. **A:** The iron bowl on the apex simulating pump encumbrance (*). **B:** The HM3 in place. **C:** The thoracic Gore Dualmesh in place (arrow). **D:** The LVAD wrapped with PTFE membrane. HM3, HeartMate3; LVAD, left ventricle assist device; PTFE, polytetrafluoroethylene.

Median sternotomy was performed. The left pleura was opened up to 2 cm from the phrenic nerve at LV apical level. To further assess implantation feasibility, we used a steel bowl simulating the LVAD pump encumbrance before heparinization (Figure 2A). On full cardiopulmonary bypass, the HM3 ring was secured to the LV apex. The heart was then fibrillated after electrical induction. Tunnelization of the driveline was performed. A full-thickness hole was made using a coring knife. At this point, the inflow cannula was inserted through the HM3 ring into the LV. Once the pump was in the correct position, the cuff lock was fully engaged, and the pump housing was rotated relative to the sewing ring and turned into its optimal, preplanned position. Figure 2B shows the pump on the heart apex. A protective film was added between the LVAD and the thoracic chest wall using the Gore Dualmesh (Gore Inc., Newark, DE) foam membrane (Figure 2C). The LVAD was wrapped with a Goretex polytetrafluoroethylene membrane (Figure 2D). The pump and proximal outflow graft were placed in the posterior left costophrenic space. Finally, the outflow graft was anastomosed to the ascending aorta. The chest was closed with stable LVAD parameters (pump flow of 2.5 L/min at 4,700 revolution per minute, pulsatility index of 5.5).

### Postoperative Course

**Figure 3.**
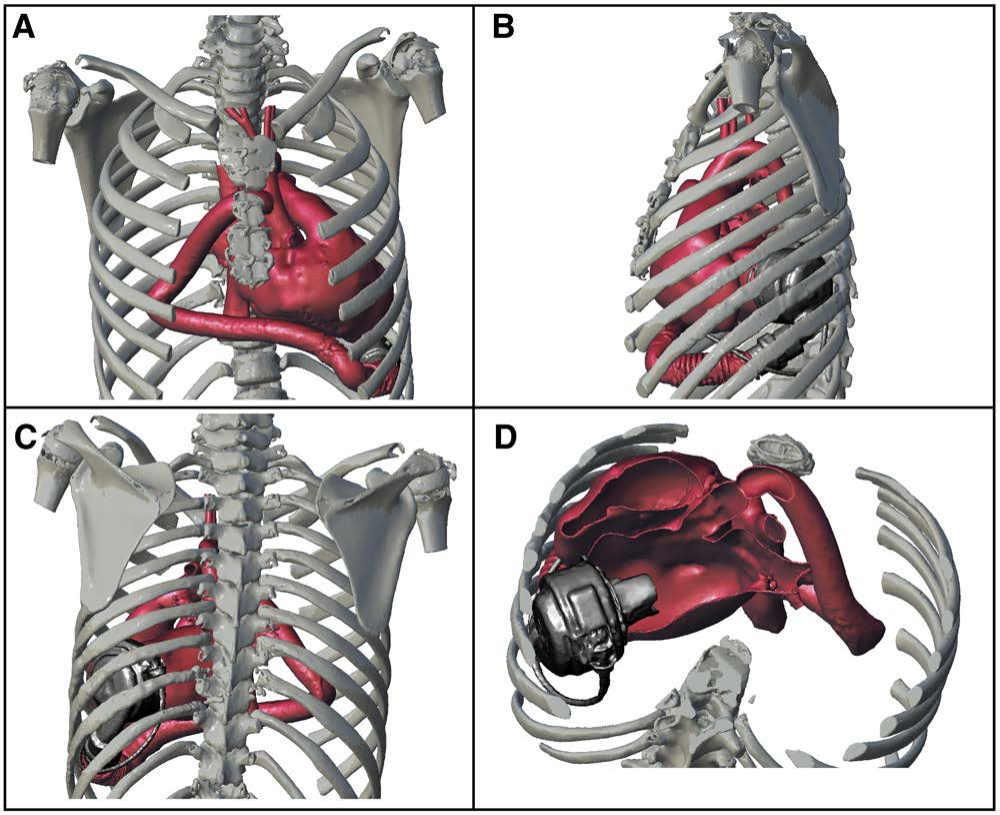
Postoperative 3D reconstructions. LVAD position after surgical implantation. **A**: Anteroposterior view. **B**: Left-lateral view. **C**: Posteroanterior view. **D**: Axial reconstruction showing inflow cannula orientation. 3D, three-dimensional; LVAD, left ventricle assist device.

The patient was transferred to the wards on postoperative day 4. The pathology was inconsistent for a myocarditis, confirming the diagnosis of primary dilated cardiomyopathy. Postoperatively, excellent HM3 functioning was registered: mean flow of 2.68 L/min ± 0.4 at a median of 4,700 revolutions per minute, mean pulsatility index of 6.75 ± 1.6, and mean pump power of 2.8 W ± 0.2. When asked, she never reported painful or “hitting-like” thoracic sensations. However, 6 days after implantation, she presented with a right hemothorax, which could be related to the start of long-term anticoagulants, because they are less manageable than intravenous unfractionated heparin. A computed tomography (CT) angiogram was performed to quantify the effusion, also allowing for a postoperative 3D reconstruction of the chest (Figure 3). On the same day, a compatible heart donor came across, and she underwent an uneventful transplant, an unexpected event given the mean waiting list time of 12 months in our region. Of note, there were no hemorrhagic traumatic lesions on the left internal chest wall.

## Discussion

We described the case of an HM3 implantation in small-sized patient. The surgical planning included cardiac-CT scans and 3D reconstructions with virtual fitting simulations. This patient-tailored approach can help to extend the use of intrathoracic LVAD also in small body sized patients.

In children weighing of less than 30 kg, the Berlin Heart EXCOR is currently the most commonly implanted LVAD, even though it requires prolonged patient hospitalization. The small chest cavity is often unable to lodge an intracorporeal LVAD without complications such as chest wall damage and inflow cannula malalignment to the interventricular septum. The preoperative 3D cardiac and rib cage reconstructions were crucial for surgical planning. Several 3D HM3 implant simulations were performed varying the HM3 position in the chest cavity. This methodology was helpful to evaluate the feasibility of LVAD lodging in the chest, allowing us to find the best location. Our simulations were adjusted considering the expected 50% reduction of LV volumes after LVAD-induced ventricular unloading, as previously mentioned. The resulting volume was used to calculate the theoretical postimplant LV longitudinal length and the consequent LVAD position.

## Conclusions

Our experience demonstrates that HM3 implantation is safe and feasible also in small patients when a complete and meticulous preoperative CT imaging with 3D simulation is applied. In fact, this methodology can help to estimate the feasibility of an LVAD implantation. This patient-tailored approach may help to further extend the pool of potential candidates to the HM3 implantation, reducing the mortality of young patient on heart transplant waiting list.

Further studies are needed to assess HM3 long-term complications in this peculiar low-BSA population.
